# In vitro Digestion of *Phaseolus vulgaris* L. Cooked Beans Induces Autophagy in Colon Cancer Cells

**DOI:** 10.3390/foods12040839

**Published:** 2023-02-16

**Authors:** Clizia Bernardi, Giulia Macrì, Marco Biagi, Elisabetta Miraldi, Federica Finetti, Lorenza Trabalzini

**Affiliations:** 1 Department of Biotechnology, Chemistry and Pharmacy, University of Siena, Via Aldo Moro 2, 53100 Siena, Italy; 2 Department of Physical Sciences, Earth and Environment, University of Siena, Via Laterina 8, 53100 Siena, Italy

**Keywords:** *Phaseolus vulgaris* L., colon cancer, autophagy, apoptosis, in vitro gastrointestinal digestion, soaking water, cooking water, common beans, tumor cell growth

## Abstract

*Phaseolus vulgaris* L. (common bean) contains high levels of proteins, unsaturated fatty acids, minerals, fibers, and vitamins, and for this reason, it represents an essential component of the diet. More than 40,000 varieties of beans have been recognized and are staple foods in the traditional cuisine of many countries. In addition to its high nutritional value, *P. vulgaris* is also characterized by its nutraceutical properties and favors environmental sustainability. In this manuscript, we studied two different varieties of *P. vulgaris*, Cannellino and Piattellino. We investigated the effects of traditional processing (soaking and cooking) and in vitro gastrointestinal digestion of beans on their phytochemical composition and anticancer activity. Using HT29 and HCT116 colon cancer cell lines, we showed that the extract obtained after gastrointestinal digestion of cooked beans (the bioaccessible fraction, BF) induces cell death through the induction of the autophagic process. We demonstrated that the BF of Cannellino and Piattellino beans at the concentration of 100 μg/mL reduces cell vitality, measured by MMT assay, of both HT29 (88.41% ± 5.79 and 94.38% ± 0.47) and HCT116 (86.29% ± 4.3 and 91.23% ± 0.52) cell lines. Consistently, the treatment of HT29 cells with 100 μg/mL of Cannellino and Piattellino BFs reduced clonogenicity by 95% ± 2.14 and 96% ± 0.49, respectively. Moreover, the activity of extracts appeared to be selective for colon cancer cells. The data shown in this work further confirm *P. vulgaris* to be among foods with beneficial effects for human health.

## 1. Introduction

Common beans *(Phaseolus vulgaris* L.) are the most essential edible legumes in the traditional cuisine of many countries in the world, playing an excellent role in human nutrition thanks to their content of proteins, unsaturated fatty acids, minerals, dietary fibers, and vitamins [1,2]. Recently, legumes have gained public attention as fundamental players in the “second green revolution” that is required to ensure food and nutritional security in the face of global climate change [3]. In this context, *P. vulgaris* plays a central role in preserving nutritional status and favoring environmental sustainability.

Extracts of several bean varieties have been investigated for their potential biological activity and possible beneficial effects on human health. The biological activity of bean extracts has been mainly attributed to the high phenolic composition that is represented by phenolic acids, hydroxycinnamic acids, flavones, flavanols, flavanones, isoflavonoids, anthocyanins, chalcones and dihydrochalcones that exert antioxidant activity [2,4]. 

In addition, several studies have indicated that the consumption of beans may prevent and control several chronic and degenerative diseases that are the main causes of mortality in the world. It has been proven that the consumption of beans is effective in reducing the risk of cardiovascular diseases (thanks to the antioxidant, anti-inflammatory, and hypolipidemic properties), ipercolesterolemy, obesity, and diabetes (thanks to the presence of α-amylase inhibitors and phytohemagglutinin and for the presence of starch) [1,2,5,6,7]. The anti-obesity and anti-diabetic activity of bean extracts has been confirmed in in vivo preclinical studies. In rat models, chronic administration of an inhibitor purified from white kidney beans reduced the levels of glycemia, food intake, and body weight without significantly altering the levels of insulin, demonstrating the hypoglycemic and anorexigenic power of this inhibitor in vivo [8]. Similarly, Neil and colleagues reported that the consumption of beans reduced abdominal fat accumulation in mice models by increasing the mass of the intestine without modification of crypts height and mucin content [9]. Interestingly, it has been also reported that in hamster models, the administration of green northern beans during a high-saturated fat diet reduces both circulating levels and the hepatic concentration of cholesterol [10].

Furthermore, it has been shown that *P. vulgaris* plays a role in the prevention of cancer [1,2], suggesting a link between a diet rich in beans and a reduced risk of numerous types of cancer, including colon (up to 47%) [11] and prostate cancer (about 22%) [12]. However, a recent meta-analysis on common beans and good health in humans suggests that cancer studies are underdeveloped, and that more in-depth studies are necessary [13]. Despite this, possible antitumoral activity has been demonstrated in animal models, in which bean administration reduced the incidence of the colon [14,15,16,17,18] and breast cancer [19,20]. In addition, by using different types of bean extracts, several researchers demonstrated that beans exert anti-proliferative, anti-inflammatory, and pro-apoptotic activities in different types of cancer cells [21,22,23,24]. However, these in vitro studies present some limitations, since the extraction methods differ considerably from the transformations that beans undergo in the digestive tract following ingestion. In this work, to better understand the impact of bean consumption on human health, we investigated two different varieties of not pigmented *P. vulgaris* by carrying out a simulated in vitro gastrointestinal digestion of cooked beans. In particular, we studied the antitumoral activity of beans soaking in water and cooking in water, and of post-digestion bioaccessible fractions, and we observed that only the latter were able to strongly reduce colon cancer cell growth by promoting autophagy.

## 2. Materials and Methods

### 2.1. Preparation of P. Vulgaris Extracts

The preparation of different extracts of *P. vulgaris* var. Cannellino and Piattellino was divided into three phases: the soaking phase, cooking phase, and in vitro digestion phase. In the first phase, 5 g of beans were soaked overnight in 100 mL of water. The soaking water (SW) was collected, and the beans were weighed again. A total of 5 g of soaked beans were then cooked in 100 mL of water, and after 3 h, the cooking water (CW) was collected. The last phase, that of in vitro digestion, involved the exposure of beans to the main digestive phases: oral, gastric, and intestinal.

Simulated digestion was performed according to the INFOGEST protocol 2.0 [25], slightly modified to obtain a higher experimental reproducibility, as published by Governa et al. [26]. Briefly, 5 g of cooked beans were mechanically whisked for 1 min in a 1% *w*/*w* NaCl solution (5 mL, pH = 4.5) containing 75 UI/mL α-amylase, thus mimicking the very brief mastication occurring in the oral cavity after the consumption of a bean puree. The bolus was then mixed with 20 mL of a solution containing gastric enzymes (pepsin, 300 UI/mL), 0.9% *w*/*w* NaCl, HCl to pH 1.8, and incubated under agitation for 2 h. The gastric chyme was diluted 2-fold in a solution with bile salts (20 mg/mL), pancreatin (activity equivalent to 4 × U.S.P., 10 mg/mL), NaHCO_3_ to pH 7, and incubated under agitation for a further 2 h. The fraction that reproduces the one absorbed by the intestinal epithelium (named the bioaccessible fraction, BF) was obtained by filtering the mixture after in vitro digestion through filter paper. The final volume of the fraction was measured after filtration [25,26]. BF samples were collected and stored at −80 °C; the solution containing digestive enzymes without beans was kept as the blank solution.

### 2.2. HPLC-DAD Analysis of Main Polyphenolic Constituents

The chemical characterization of polyphenolic profile of CW and BF samples was performed through HPLC-DAD analysis using a Shimadzu Prominence LC 2030 3D instrument equipped with a Bondapak^®^ C18 column, 10 µm, 125 Å, 3.9 mm × 300 mm column (Waters Corporation, Milford, MA, USA) as described in [22]. Water solutions containing 0.1% (*v*/*v*) formic acid (A) and 0.1% (*v*/*v*) acetonitrile (B) were used as the mobile phase. The following program was applied: B from 10% at 0 min o 25% at 15 min, then B 35% at 25 min, B 50% at 30 min, and B 10% at 32 min holding this parameter until 35 min; flux was set at 0.85 mL/min. Chromatograms were recorded at 254, 280, 330, and 350 nm. Analyses were performed using 10 µL of samples; gallic acid, chlorogenic acid, caffeic acid, catechin, genistein, daidzein, quercetin, and kaempferol (Sigma-Aldrich) were used as external standards. Calibration curves were established using reference standards ranging from 0.008 mg/mL to 0.500 mg/mL. The correlation coefficient (R2) of each curve was >0.99 [22]. 

Compounds identified through the retention time and UV-vis spectrum in comparison with reference standards were quantified according to the peak areas and by interpolating data from standard curves. With regard to total polyphenols and total hydroxycinnamic derivatives, the quantification was performed by summing peak areas of all compounds revealed by using this specific method to have a characteristic UV-vis spectrum (λmax at 255–280 nm for all phenolics and λmax at 255–280 nm and 320–330 nm for hydroxycinnamic derivatives). Quantification of total polyphenols was carried out using gallic acid as standard, and that of total hydroxycinnamic derivatives using chlorogenic acid as standard.

### 2.3. Cell Culture

HT29, colorectal adenocarcinoma cells (ATCC, Rockville, MD, USA) were cultured in RPMI-1640 (Euroclone) medium supplemented with 10% fetal bovine serum (FBS, EuroClone), 100 U/mL penicillin/streptomycin (Euroclone), and 4 mM L-glutamine (Euroclone). HF, human fibroblasts cells (ATCC, Rockville, MD, USA), HCT116 colorectal carcinoma cells (ATCC, Rockville, MD, USA), and HaCaT, human epidermal keratinocytes, were cultured in DMEM with 4500 mg/L glucose (Euroclone) supplemented with 10% FBS, 100 U/mL penicillin/streptomycin, and 4 mM L-glutamine. All cell lines were grown at 37 °C and 5% CO_2_.

### 2.4. MTT Assay

Either 3.5 × 10^3^ (HT29), 2.5 × 10^3^ (HCT116), 3.0 × 10^3^ (HaCaT), or 1.5 × 10^3^ (FU) cells/well were seeded in 96-multiwell plates in medium with 10% FBS, and after adherence were maintained for 24 h in medium containing 0.1% serum. After 24 h, cells were treated with different concentrations (1, 10, and 100 µg/mL) of Piattellino and Cannellino bean samples (SW, CW, BF), and the blank solution. After 48 h, the medium was removed, and cells were incubated for 4 h with fresh medium in the presence of 1.2 mM MTT (3-(4,5-dimethylthiazol-2-yl)-2,5-diphenyltetrazolium bromide) (Sigma-Aldrich). Then, the supernatant of each well was removed and 50 µL of dimethyl sulfoxide was added into the well to dissolve the blue formazan crystals. Cell viability was evaluated by measuring the absorbance at 595 nm using a microplate reader (EnVision, PerkinElmer, Waltham, MA, USA). Data were expressed as a percentage of the basal control.

### 2.5. Western Blotting Analysis

HT29 (3.5 × 10^5^ cells/well) were seeded in 6-well multi-plates in medium with 10% serum. After 24 h, cells were starved for 24 h in medium containing 0.1% serum, and then treated with BF (1, 10, 100 µg/mL) or the blank solution. After 48 h, extraction of total proteins was performed by lysing cells in precooled radioimmunoprecipitation assay (RIPA) buffer. Cell lysates were centrifuged at 13,000× *g* for 15 min at 4 °C and protein concentration was determined using the BCA method (BCA protein assay kit, Euroclone). Subsequently, equal amounts of proteins (50 μg) were treated with Laemmli buffer, boiled for 7 min, separated by 10% sodium dodecyl sulphate (SDS)-polyacrylamide gel electrophoresis (PAGE), and transferred onto a nitrocellulose membrane using a Semidry Electro-blotter System (Galileo Bioscience, Cambridge, MA, USA). Unspecific protein-binding sites were blocked by incubation with 5% milk and 0.5% Tween-20 in Tris-buffered saline (TBS) at room temperature for 1 h, and membranes were then incubated overnight at 4 °C with appropriate dilutions of anti-LC3 or anti-βtubulin (Cell Signaling Technology) primary antibodies. Subsequently, membranes were incubated for 1 h with secondary antibodies (Sigma-Aldrich). Immunoreactive proteins were then visualized by an enhanced chemiluminescence detection system (Euroclone) and images were digitalized with Image Quant LAS4000 (GE Healthcare Europe GmbH, Milano, Italy). Immunoreactive bands from Western blots were quantified by densitometry using the ImageJ software (open-source image processing program, National Institutes of Health, Bethesda, MD, USA).

### 2.6. Clonogenic Assay

HT29 cells were plated in 24 multi-well plates (350 cells/dish) in a medium containing 10% FBS (Euroclone, Milano, Italy). After 24 h, cells were treated with Cannellino and Piattellino SW, CW, and BF samples (1–10–100 μg/mL) in 1% FBS (*v*/*v*) and kept for 10 days. Colonies were fixed with methanol, stained, and counted (>50 cells) [27]. 

### 2.7. Trypan Blue Assay

HT29 cells (7.5 × 10^5^ cells/mL) were suspended in RPMI 10% FBS (Euroclone) and treated with Cannellino or Piattellino BF (100 ug/mL) or with the blank solution. After 48 h, one part of cell suspension was mixed with one part of 0.4% trypan blue in a microtube and counted with LUNA-II Automated Cell Counter (Logos Biosystems).

### 2.8. Immunofluorescence Assay

HT29 cells (7.5 × 10^4^ cells/well) were seeded on glass coverslips that wereplaced into 24 multi-well plates and maintained in RPMI 10% FBS for 24 h. Cells were treated with Cannellino or Piattellino BF (100 µg/mL) and, after 24 h, fixed in acetone for 5 min. After blocking with 3% bovine serum albumin (BSA) for 1 h, cells were stained with the anti-LC3B antibody overnight at 4 °C. Samples were then incubated 1 h with fluorescein (FITC)-conjugated anti-rabbit (1:150, Alexa Fluor 488, ThermoFisher) and for 5 min with DAPI (0.1 µg/mL, cell signaling). Cells were examined with an ECLIPSE Ts2 microscope and images were obtained with NIS-Elements software (Nikon).

### 2.9. Annexin V-FITC Staining

HT29 cells (7.5 × 10^4^ cells/well) were seeded on glass coverslips placed into 24 multi-well plates. After 24 h, cells were treated with Cannellino or Piattellino BF (100 µg/mL) for 18 h. Each well was washed with PBS and incubated with 1X Annexin V Binding Buffer containing 1 µL of Annexin V-FITC conjugated antibody and 12.5 µL of propidium iodide (cell signaling) for 10 min on ice in the dark. Cells were fixed with 4% paraformaldehyde and analyzed by an ECLIPSE Ts2 microscope and NIS-Elements software (Nikon).

### 2.10. DAPI

Cells were seeded in a 96-well plate with a density of 3.5 × 10^3^/well in RPMI containing 10% of serum. After 18 h of treatment with Cannellino or Piattellino BF (100 µg/mL), cells were fixed with 4% paraformaldehyde and incubated with 0.1 µg/mL DAPI (cell signaling) for 5 min at room temperature in the dark. Cells were washed in PBS and analyzed by an ECLIPSE Ts2 microscope and NIS-Elements software (Nikon).

### 2.11. Senescence Assay

HT29 cells were seeded in 24 multi-well plates (7.5 × 10^4^ cells/well) in RMPI medium with 10% FBS. After adherence, cells were treated with Cannellino or Piattellino BF (1, 10, and 100 µg/mL) for 24 h. Senescence was determined using a senescence β-galactosidase staining kit (cell signaling). Briefly, cells were washed with PBS, fixed with fixative solution, and incubated with β-galactosidase staining solution at pH 6.0, 37 °C, overnight in a dry incubator. While the β-galactosidase was still on the plate, cells were checked under an ECLIPSE Ts2 microscope, and images were obtained using NIS-Elements software (Nikon).

### 2.12. Statistical Analysis

Data were generated from independent experiments and expressed as mean ± standard deviation (SD). Statistical analysis was performed using Student’s t-test, a one-way ANOVA, or Tukey’s multiple comparisons test (GraphPad Prism). Differences in a dataset of *p* < 0.05 were considered statistically significant. 

## 3. Results

### 3.1. Chemical Analysis of P. Vulgaris Extracts

Chemical analyses of polyphenols contained in SW, CW, and BF extracts of Cannellino and Piattellino bean varieties showed that a not negligible content of polyphenols was found in CW and BF, whereas the soaking procedure at room temperature (SW) did not provide a detectable yield of this class of secondary metabolites.

**Figure 1:**
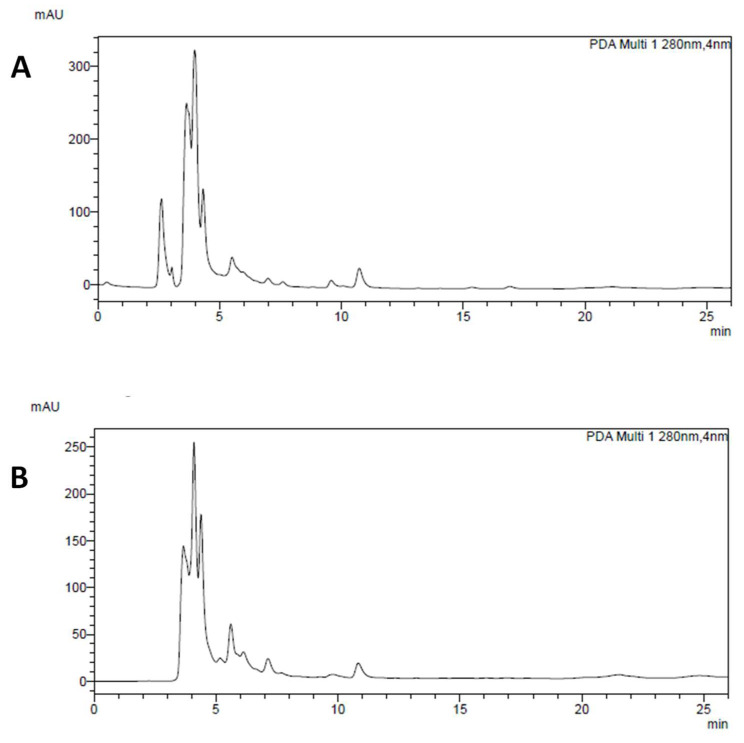
Chromatogram of Cannellino (**A**) and Piattellino (**B**) CW. Gallic acid (RT = 4.5 min) is the main phenolic acid, whereas chlorogenic acid (RT = 10.8 min) is the most present hydroxycinnamic derivative. At RT = 5.5, it is present another phenolic acid, while at RT = 6.2, 7.1, 9.9, 15.5 and 17.1 min, other hydroxycinnamic derivatives are present but not identifiable through HPLC-DAD.

As shown in Table 1, the chemical profile of CW extracts of Cannellino and Piattellino was very similar. According to what was previously reported about the similar variety of not pigmented Fagiola di Venanzio bean [22], Cannellino and Piattellino mainly contained hydroxycinnamic derivatives (HCD), identified by means of diagnostic UV spectra (λmax at 255–280 nm and 320–330 nm), and simple phenolic acids (PA, λmax at 255–280 nm at low retention times). Gallic acid (RT = 4.5 min) and chlorogenic acid (RT = 10.8 min) were the most represented PA and HCD in both CWs, respectively. The chromatograms of Cannellino and Piattellino CW recorded at 280 nm are reported in Figure 1A,B.

**Figure 2:**
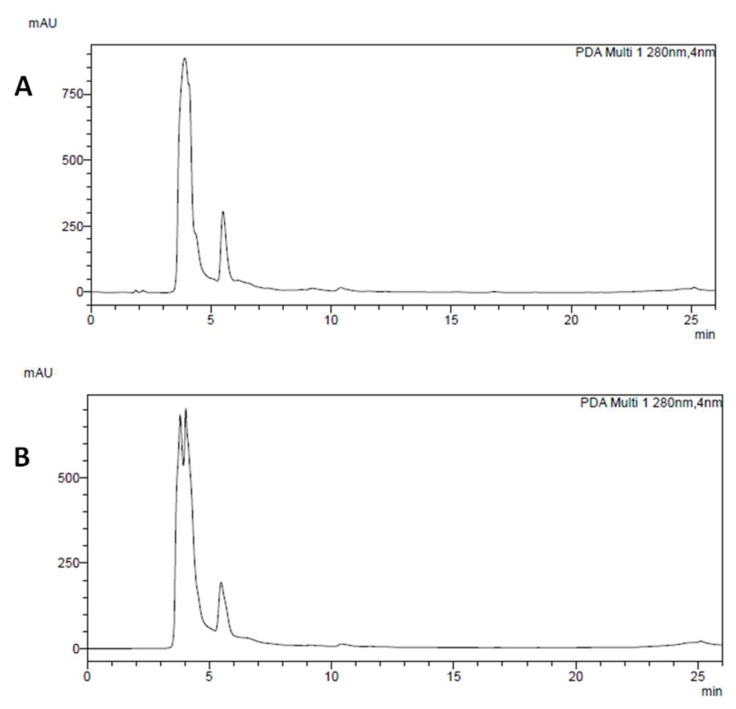
Chromatogram of Cannellino (**A**) and Piattellino (**B**) BF. The main compounds that could be identified in the chromatogram are hydroxycinnamic derivatives, namely those at RT = 9.2 and 10.4 min in good amounts in Cannellino, and minority compounds at RT = 15.3 and 16.8 min.

In bioaccessible fractions obtained after the simulated gastrointestinal digestion (BF), it was very difficult to identify single polyphenols because of the matrix interference of the digestive mixture. For this reason, it was not possible to perform PA and gallic acid quantification. On the other hand, HCD were clearly identified in BF extracts (Figure 2A,B), and we found that the content of these compounds was twice greater in Cannellino than in Piattellino. Native chlorogenic acid was completely lost after the digestive process both in Piattellino and in Cannellino samples. 

The predominant compound in chromatograms of beans after simulated digestion was not present in pre-digested beans; the close RT compared to intact chlorogenic acid (RT = 10.4 vs. 10.8) and a very similar UV spectrum suggested that this compound could be identified as a chlorogenic acid derivative. Other HCD found after the simulated digestion (RT = 9.2, 15.3, and 16.8 min), may most likely be derivatives of HCD already found in beans analyzed before the digestive process.

### 3.2. Effects of P. Vulgaris Extracts on Cell Vitality and Growth of Colon Cancer Cells

**Figure 3:**
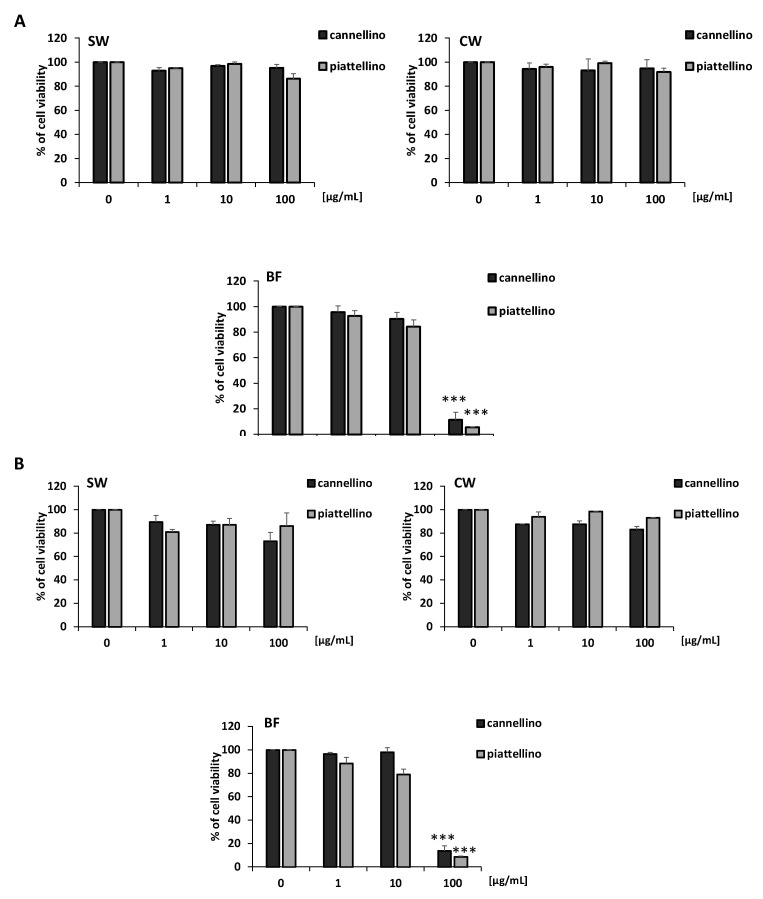
The bioaccessible fraction of cooked beans (BF) inhibits colon cancer cell vitality. Cell vitality was measured in the presence or absence of Cannellino and Piattellino extracts (1, 10 and 100 µg/mL) by MTT assay. HT29 (**A**) and HCT116 (**B**) cells were exposed to treatments for 48 h. Data are reported as % of cell viability and are the means of three experiments run in triplicate. *** *p* < 0.0001 vs. basal.

To evaluate the potential antitumoral activity of SW, CW, and BF extracts of the two *P. vulgaris* varieties, we performed an MTT assay on HT29 and HCT116 cell lines. Colon cancer cells were treated with increasing concentrations of Cannellino and Piattellino preparations and fractions (1, 10, and 100 µg/mL), and with the blank solution for 48 h. Results reported in Figure 3A,B indicate that soaking water (SW) and cooking water (CW) did not modify cell vitality, while the bioaccessible fraction (BF) at the concentration of 100 µg/mL dramatically reduced cell vitality. This effect was not related to the enzymes used in the in vitro gastrointestinal digestion of beans, since the blank solution did not show activity, with a light reduction of HT29 cell vitality (16%) at the highest concentration (data not shown).

**Figure 4:**
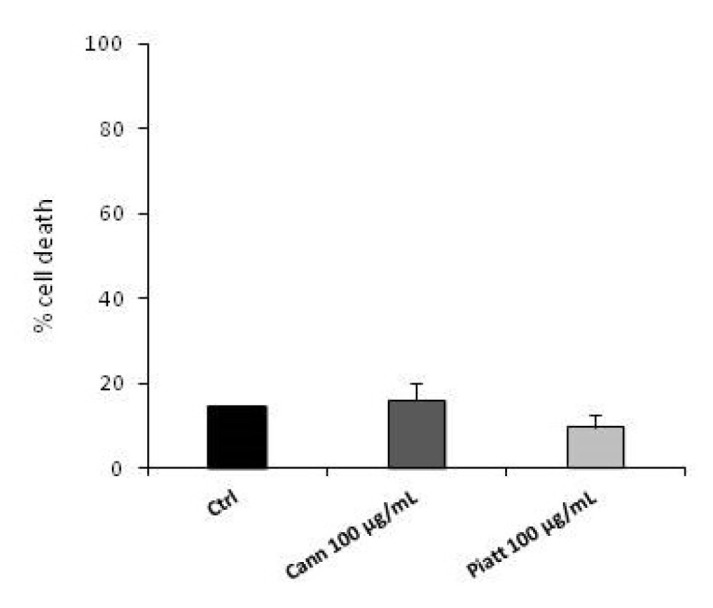
BF does not induce cell death. Cell toxicity of Cannellino and Piattellino BF (100 µg/mL) was evaluated by trypan blue assay. HT29 cells were exposed to treatments for 48 h, and dead cells were labeled by using the trypan blue reagent. Blue cells were counted using a LUNA-II Automated Cell Counter (Logos Biosystems). Data are reported as % of cell death and are the means of three experiments run in triplicate. Ctrl: untreated cells.

**Figure 5:**
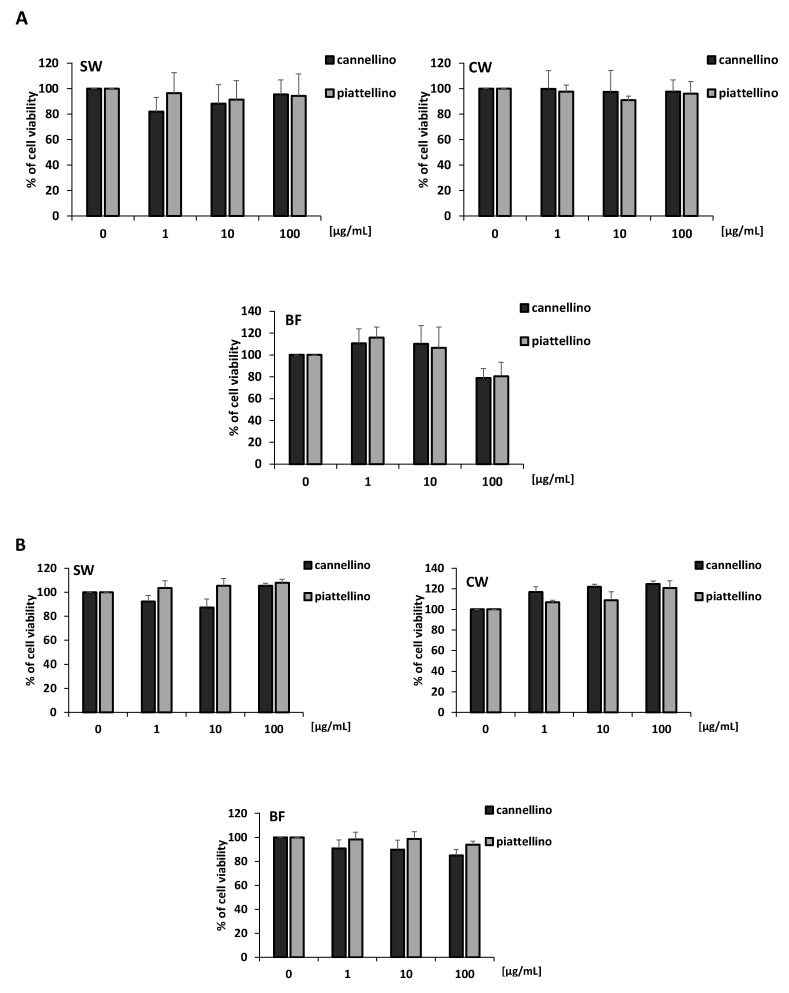
BF does not affect human fibroblasts and Hacat cells’ vitality. Cell vitality was measured in the presence or absence of Cannellino and Piattellino BF (1, 10, and 100 µg/mL) by MTT assay. HF (**A**) and HaCaT (**B**) cells were exposed to treatments for 48 h. Data are reported as % of cell viability and are the means of three experiments run in triplicate.

In addition, in order to exclude that the reduction in cell vitality was related to nonspecific BF toxicity, we performed a trypan blue assay. As shown in Figure 4, Cannellino and Piattellino BF did not induce cell death in the HT29 cell line. Moreover, the effect of BF appeared specific for cancer cells, since the treatment for 48 h of human fibroblasts (HF) and keratinocytes (Hacat) with BF fractions did not affect their vitality (Figure 5A,B).

**Figure 6:**
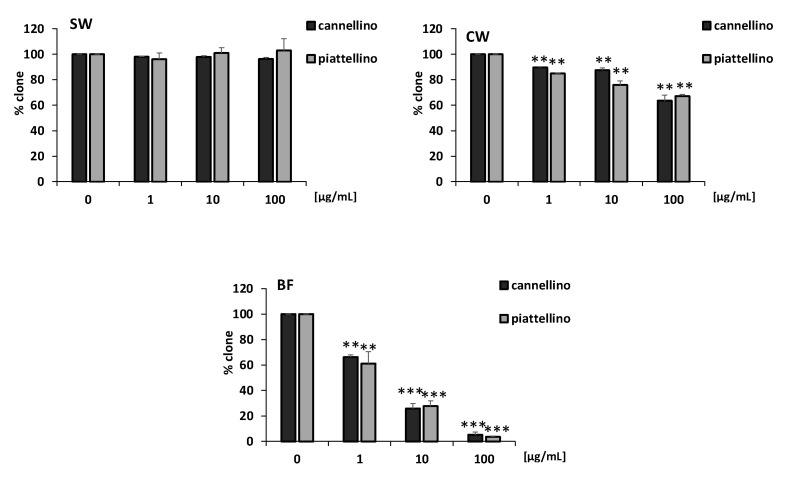
BF reduces HT29 cell clonogenicity. Percentage of colonies of HT29 cells in response to different concentrations (1, 10, and 100 µg/mL) of SW, CW, and BF. Data are expressed as % over basal control and are representative of three independent experiments run in triplicate. Statistical analysis: *** *p* < 0.001, and ** *p* < 0.01 vs. basal condition.

To confirm the anti-proliferative role of BF fractions of beans, we performed a clonogenic assay. HT29 cells were incubated for 10 days with different concentrations (1, 10, and 100 µg/mL) of Cannellino and Piattellino SW, CW, and BF samples. Experimental outcomes showed that BF was able to significantly restrain the colony formation of colon cancer cells also at the lowest concentration tested (Figure 6). Interestingly, CW also reduced colony formation, albeit to a lesser extent. 

These data suggest that the BF of cooked Cannellino and Piattellino beans selectively regulate colon cancer cell vitality and growth without nonspecific cell toxicity.

### 3.3. Bioaccessible Fraction of P. Vulgaris Beans Promotes Colon Cancer Cell Death through the Activation of Autophagy

To further investigate the antitumor mechanisms of *Phaseolus* extracts against colon cancer cells, we explored the effects of Cannellino and Piattellino bioaccessible fractions on apoptosis, senescence, and autophagy processes. 

**Figure 7:**
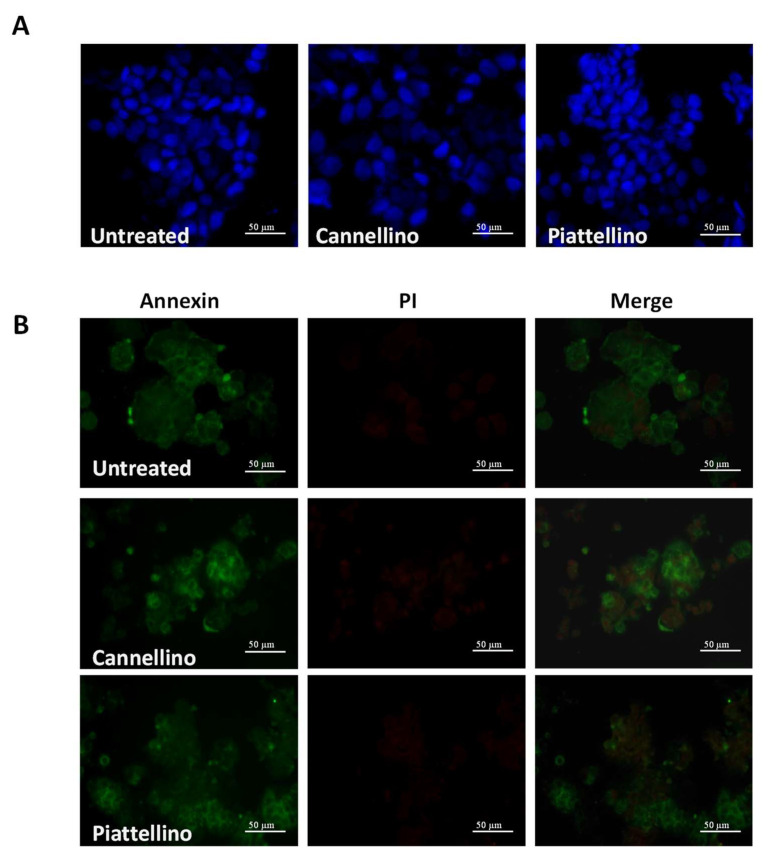
BF does not induce colon cancer cell death through the activation of apoptotic process. HT29 cells were incubated with 100 µg/mL of Cannellino and Piattellino BF and stained with (**A**) 0.1 µg/mL DAPI or (**B**) Annexin V-FITC conjugated antibody and Propidium Iodide. Data are representative of three independent experiments.

Previous reports indicate that extracts from different varieties of *P. vulgaris* can promote apoptosis of melanoma and colon cancer cells [20,21]. To verify whether our extracts induced cell apoptosis, HT29 cells were treated for 18 h with 100 µg/mL of Cannellino and Piattellino BF, and DAPI staining was applied to evaluate nuclear changes that occur during the apoptotic process. As evident in Figure 7A, both extracts did not modify nuclear integrity. Similarly, immunostaining with annexin V and propidium iodide (PI) indicated that neither the apoptotic process not the necrotic process was activated by BF (Figure 7B).

**Figure 8:**
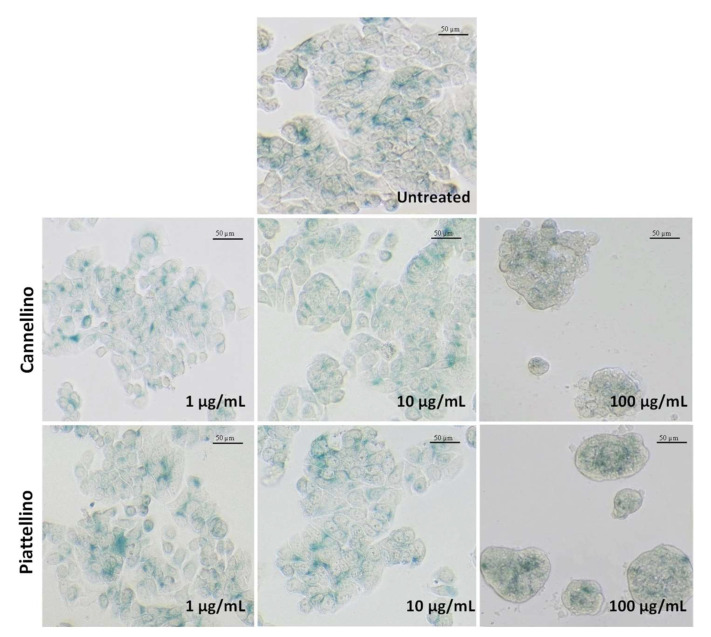
Effects of Cannellino and Piattellino BF on cellular senescence. HT29 cells were treated with Cannellino and Piattellino BF (1, 10, and 100 µg/mL) for 24 h. The figure reports images of cells after staining for β-galactosidase activity, with the blue color representing senescent cells. Data are representative of three different experiments.

In light of these data, we then evaluated whether cancer cells undergo a senescent phenotype when treated with BF. In fact, senescence is considered a highly dynamic process associated with different cellular changes and distinct phenotypic alterations including a stable and generally irreversible proliferation arrest. Interestingly, several studies have shown that treatment with some anticancer agents may induce senescence in tumor cells, while no data are available for *P. vulgaris* to date [28]. To verify this hypothesis, we treated the HT29 cell line with increasing concentrations of BF (from 1 to 100 µg/mL) and we performed β-galactosidase staining. As shown in Figure 8, BF did not induce cellular senescence.

**Figure 9:**
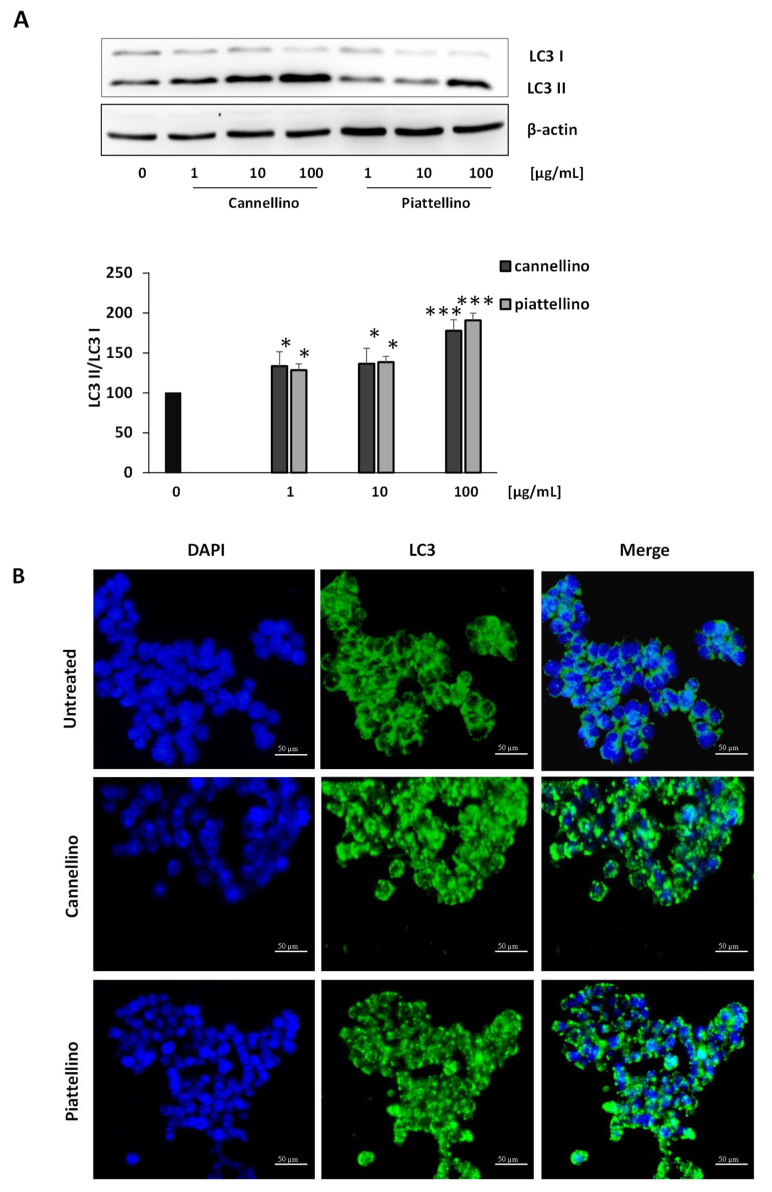
Cannellino and Piattellino BF promotes the activation of autophagy in colon cancer cells. (**A**) HT29 cells were treated with increasing concentrations of BF, and LC3 I and LC3 II were detected through Western blotting. Images are representative of three different experiments. Quantification of bands was performed by ImageJ software. *** *p* < 0.001, * *p* < 0.05 vs. basal conditions. (**B**) HT29 cells were seeded on glass coverslips and treated with Cannellino and Piattellino BF (100 µg/mL) for 24 h, fixed, and stained with anti LC3 antibody. Images are representative of two different experiments.

We then investigated if *Phaseolus* extracts were able to induce the autophagic process. In fact, autophagy is a self-eating process used by eukaryotic cells to degrade their cytoplasmic proteins and damaged organelles under the control of autophagy-related genes and proteins [29]. To examine the effects of BF on autophagy, we treated HT29 cells with increasing concentrations (1, 10, and 100 µg/mL) of bean extracts for 24 h, and we analyzed LC3 I and LC3 II modifications by Western blotting and immunofluorescence assay. The results revealed that both Cannellino and Piattellino BF significantly increased LC3 II protein formation measured by Western blotting (Figure 9A), and that diffuse green spots observed in the control cells after LC3 staining became evident green autophagy spots in the BF-treated cells (Figure 9B). The blank solution was unable to induce LC3 II (data not shown). These data clearly indicate that the bioaccessible fraction of Cannellino and Piattellino varieties of *P. vulgaris* beans can promote colon cancer cell death through the activation of autophagy.

## 4. Discussion

*Phaseolus vulgaris* beans are regarded as highly nutritional foods known to have beneficial effects on human health. Many studies have provided evidence to support the hypothesis that beans may be protective against different health issues, and the consumption of beans has been related to the prevention and control of numerous chronic and degenerative diseases, including cardiovascular diseases, obesity, diabetes, and cancer [1,2]. 

Colon cancer is one of the leading causes of death worldwide. Colon cancer onset and progression have been mainly linked to ambiental factors, including environmental and food-borne mutagens, specific intestinal pathogens, and chronic intestinal inflammation, rather than heritable genetic changes. Epidemiologic studies have suggested that a diet rich in legumes and particularly in beans may protect from this type of cancer [5,30]. This hypothesis has been confirmed by in vivo studies. By using azoxymethane (AOM)-induced rats, Vergara-Castañeda et al., showed that the non-digestible fraction of cooked beans (*P. vulgaris*) suppresses the formation of colonic aberrant foci (CAC) and modulates the expression in the colon tissue of numerous genes involved in apoptosis, cell-cycle regulation and arrest, inhibition of proliferation and inflammation, and DNA repair [16,31]. The gene expression studies were then confirmed by Feregrino-Pérez et al., who reported that the non-digestible fraction of cooked beans inhibits colon carcinogenesis at an early stage by inducing cell cycle arrest of colon cells and morphological changes linked to apoptosis [17]. All these data support the chemoprotective effect of common beans on early-stage colon cancer.

However, a very recent systematic review and meta-analysis of clinical and randomized controlled trials on *P. vulgaris*’ effects on human health suggest that cancer studies may be underdeveloped, and may not demonstrate whether common beans reduce the likelihood of developing cancers [13]. These observations suggest that large-scale intervention trials must be performed to correlate consumption of beans with human health and encourage more in-depth studies. 

A critical point in studies on bean activity may be represented by the lack of physiological conditions of extract preparation, particularly for studies in in vitro cellular models. In fact, understanding the physiological response to specific foods requires knowledge of the complex digestive processes within the human digestive tract. These studies may allow us to obtain more accurate information about the active molecules and evaluate the molecular signaling pathways involved in bean anticancer activity.

Considering these aspects, in vitro models have been used to simulate the digestion of foods. In vitro methods simulating digestion processes are widely used to study the gastrointestinal behavior of foods and have the advantage of being able to process many samples, being more rapid and less expensive than in vivo models. In vitro digestion methods include the oral, gastric, and small intestinal phases, and rarely, large intestinal fermentation. These methods consider the presence of digestive enzymes, pH, digestion time, and salt concentrations, thus mimicking physiological conditions. 

Here, we evaluated the bioactivity of extracts derived from specific bean preparations by mimicking in vitro the traditional cooking procedures of beans (soaking and cooking in water) and their digestion by the gastrointestinal tract. We determined the chemical composition of different extracts of Cannellino and Piattellino beans in terms of polyphenol content and anticancer activity by using different biological/pharmacological approaches. Interestingly, we found that soaking and cooking water was not active in all the conditions tested. These data were in line with Ombra et al., who showed that acetone extracts of non-pigmented beans were inactive against cancer cells [21]. As opposed to the soaking and cooking water, the extracts obtained following in vitro digestion of cooked beans showed a strong specific antiproliferative activity on colon cancer cells by inducing the activation of the autophagic process. Importantly, this activity seemed to be specific to cancerous cells.

The anticancer activity of the bioaccessible fraction of cooked beans, not possessed by the other bean extracts (soaking water and cooking water), can be attributed to the different chemical composition of the fractions, and more specifically to the presence of specific molecules generated following the digestive process. These observations are in line with Mojica et al., who described the biological activities of small peptides obtained from the pepsin/pancreatin digestion of four varieties of *P. vulgaris* beans [32]. They showed that protein isolates after simulated gastrointestinal digestion of common beans possess significant antioxidant, antidiabetic, and antihypertensive potential properties. It appears evident that small peptides derived from beans may interact with specific amino acid residues in the catalytic site of some enzymes, as reported for dipeptidyl peptidase-IV (DPP-IV), Angiotensin-converting enzyme (ACE) and α-glicosidase, which are involved in the regulation of functions important for cardiovascular homeostasis [32,33,34,35].

Our data regarding the chemical composition of different bean extracts, currently limited to the determination of the polyphenol content, show that after in vitro gastrointestinal digestion, HCD represents the most retained polyphenolic fraction, while native chlorogenic acid was completely lost. Thus, due to amylase and protease activity and pH change, the bioaccessible fraction of cooked beans probably contains modified polyphenols, but also degraded complex sugars and oligopeptides.

The partial chemical analysis reported here has been limited by inevitable analytical interferences after simulated digestion; however, the aim of this work was to know as much as possible about the polyphenolic composition of samples that have been assayed in biological tests. Investigation of the chemical profile of a digested phytocomplex is a current challenge that the scientific community is trying to face through sophisticated methods such as metabolomic analysis, and was not within the scope of this work whose development will have as main objective the investigation of the chemical profile of the digested phytocomplex in order to identify the molecules responsible for anticancer activity.

## 5. Conclusions

In this manuscript, we analyzed the activity of two common varieties of *P. vulgaris* against colon cancer cells. 

We used *Phaseolus* extracts obtained by mimicking the traditional cooking procedures of beans (soaking and cooking in water) and their digestion by the gastrointestinal tract (i.e., the bioaccessible fraction). We showed that the bioaccessible fraction induces the death of cancer cells through the induction of the autophagic process, while the soaking and cooking water were inactive. Moreover, the activity of the bioaccessible fraction appeared to be selective for colon cancer cells.

Overall, this study supports the beneficial effect on human health that may be derived from the presence of beans in the diet and stimulates research into the molecules responsible for these health properties. Thus, beans, typically known as cheap foods easily accessible to the population, play a key role as nutraceuticals, showing healthy activities and arousing considerable interest in the scientific community, which places them at the center of numerous studies.

## Figures and Tables

**Table 1:** Polyphenol content of CW and BF extracts of Cannellino and Piattellino beans.

Components	Quantification (mg/g)			
	Piattellino		Cannellino	
	CW	BF	CW	BF
Total polyphenols	0.75 ± 0.03	n.d.	0.75 ± 0.03	n.d.
Total hydroxycinnamic derivatives	0.21 ± 0.01	0.10 ± 0.01	0.27 ± 0.02	0.20 ± 0.04
Gallic acid	0.39 ± 0.03	n.d.	0.34 ± 0.03	n.d.
Chlorogenic acid	0.08 ± 0.01	0	0.12 ± 0.01	0

## Data Availability

All data included in this study are available by contacting the corresponding authors.
